# Recycled PMMA particles from milled discs in denture base materials for improved sustainability

**DOI:** 10.1186/s12903-025-05943-1

**Published:** 2025-04-25

**Authors:** Zinab Hijazi, Abubaker Qutieshat, Chibuzo Nlemorisa, Abdurahman Salem

**Affiliations:** 1 Centre for Dental Sciences, School of Health & Human Sciences, University of Bolton, Bolton, UK; 2 Restorative Dentistry, Oman Dental College, Muscat, Oman; 3 Restorative Dentistry & Dental Biomaterials, Dundee Dental Hospital & School, University of Dundee, Dundee, UK

**Keywords:** Dental materials, Mechanical properties, Polymethyl methacrylate, Recycling, Sustainability

## Abstract

**Objectives:**

Recycling polymethyl methacrylate (PMMA) in dentistry offers a sustainable solution to address the environmental challenges posed by material waste. This study evaluates the feasibility of incorporating recycled (PMMA) particles into heat-cured denture base materials, focusing on the effects of particle size and concentration on flexural strength, surface hardness, and surface roughness.

**Material and Methods:**

Recycled PMMA particles, sourced from milling residuals, were classified into fine (< 400 μm) and whole particles and incorporated into virgin PMMA at 10% and 20% weight/weight (w/w) concentrations to form five groups: Control (100% virgin PMMA), Fine 10% (90% virgin PMMA + 10% fine particles), Fine 20% (80% virgin PMMA + 20% fine particles), Whole 10% (90% virgin PMMA + 10% whole particles), and Whole 20% (80% virgin PMMA + 20% whole particles). Flexural strength (*n* = 10) was evaluated using a three-point bending test, surface hardness (*n* = 10) was assessed using the Vickers hardness test (VHN), and surface roughness (*n* = 10) was measured using an optical profilometer. Statistical analysis was performed using two-way ANOVA and Tukey’s post hoc test, with *p* < 0.05 considered statistically significant.

**Results:**

The results showed that fine particle groups (10% and 20%) maintained flexural strength comparable to the control, whereas whole particle groups exhibited significantly lower values (*p* < 0.05). Surface hardness improved in fine particle groups, with the highest value in the 20% fine particle group, whereas whole particles, particularly at 20%, resulted in the lowest hardness (*p* < 0.05). Surface roughness remained unaffected across all groups, with values staying within clinically acceptable limits (Ra < 0.2 μm).

**Conclusions:**

These findings suggest that fine recycled PMMA particles can be effectively integrated into denture base materials to maintain or improve specific properties while contributing to sustainability. Clinical Relevance: This study underscores the potential of recycled PMMA as an environmentally friendly alternative, aligning with global sustainability goals in dental material development.

## Introduction

The healthcare sector, including dentistry, is a significant contributor to environmental waste, producing considerable quantities of single-use plastics, packaging, and residual materials. Among these, polymethyl methacrylate (PMMA) is extensively used in dental prosthetics, such as dentures and orthodontic appliances, due to its adaptability, durability, and ease of use. However, improper disposal of PMMA has adverse environmental implications, including contributions to greenhouse gas emissions and microplastic pollution [1].

Recognizing the environmental challenges posed by materials like PMMA, the focus on sustainability in dentistry has seen exponential growth in recent years. A bibliometric analysis conducted using Scopus, with the search key [TITLE-ABS-KEY ( sustainab* ) AND ( LIMIT-TO ( SUBJAREA, “DENT” ) )], identified 851 publications addressing sustainability in dentistry. Of these, 114 were published in 2023, while this number more than doubled to 236 in 2024 alone. This surge reflects the increasing global emphasis on sustainability driven by the adoption of the United Nations’ Sustainable Development Goals (SDGs) by academic institutions and research platforms [2]. These goals encourage innovative strategies to minimize waste and enhance the sustainability of healthcare practices.

In 2018, Times Higher Education (THE) announced a new global ranking system measuring universities’ success in delivering the UN’s SDGs, which in turn facilitated the launch of the THE Impact Rankings in 2019. This initiative encouraged universities to align their research with sustainability goals, increasing the visibility and impact of such work. Additionally, Scopus began mapping research publications to SDGs in 2022, further enhancing the prominence of sustainability-focused studies. These efforts have likely incentivized researchers and institutions to prioritize sustainability-related topics, including recycling strategies for dental materials, explaining the sharp rise in publications observed in recent years^3–6)^.

PMMA is a versatile thermoplastic polymer widely used in dentistry due to its mechanical properties, transparency, and cost-effectiveness. Its applications range from dentures and orthodontic appliances to maxillofacial prosthetics. The ISO 20795-1:2013 standard mandates that PMMA materials must meet specific mechanical thresholds, such as a minimum flexural strength of 65 megapascal (MPa) [7]. Despite its advantages, PMMA’s manufacturing and disposal contribute significantly to environmental pollution. Approximately 15 million dentures are produced annually in the UK alone, with most PMMA waste left unrecycled [8].

The production of PMMA involves high energy consumption and generates substantial greenhouse gas emissions. For instance, the polymerization of methyl methacrylate (MMA) into PMMA requires 116.23 megajoule (MJ) per kilogram and emits 5.9 kg (kg) of CO_2_[9]. Improper disposal of PMMA waste exacerbates the issue, with microplastics harming marine ecosystems and posing health risks to humans [10]. Recycling PMMA, while technically feasible, remains underutilized due to the lack of industry guidelines and standardized methodologies.

The growing emphasis on sustainability in dentistry provides a timely impetus for addressing these issues, focusing on innovative recycling strategies to reduce waste while maintaining material integrity. Several studies highlight the potential of recycling dental materials to reduce environmental impact and the viability of recycling PMMA for sustainable dental applications. For example, it was demonstrated that recycled PMMA could be reintegrated into dental base resins without compromising key mechanical properties [11]. Similarly, Improvements were observed in surface hardness when recycled acrylic resin was incorporated into PMMA mixtures [12].

Other studies have also investigated the incorporation of recycled components such as polyetheretherketone into PMMA, noting improvements in both surface hardness and roughness [13]. Furthermore, it has been shown that PMMA retains its advantageous properties through multiple recycling cycles, making it a highly feasible material for sustainable reuse [14]. Despite these findings, the literature emphasizes the lack of standardized guidelines or frameworks for integrating recycled PMMA into dental materials, particularly in industrial or clinical settings.

The cost-saving potential of using recycled PMMA in dental laboratories was highlighted in the literature with estimates suggesting a reduction of up to 50% in raw material costs when incorporating 50% recycled PMMA into the mixture [11]. However, challenges such as ensuring consistent material properties and the technical feasibility of recycling processes remain underexplored, representing critical areas for further research. Furthermore, there is limited data on how particle size and concentration influence the performance of recycled PMMA in denture bases. This study aims to fill this gap by evaluating the mechanical and surface properties of heat-cured PMMA with varying sizes and percentages of recycled particles, providing insights into the feasibility of sustainable PMMA recycling.

## Materials and methods

This study was approved by the Oman Dental College Ethics Committee, ensuring compliance with ethical standards for research. The methodologies adhered to ISO standards for dental materials testing (i.e., ISO 20795-1:2013 and ISO 178:2019).

The experiment examined two independent variables: the percentage of recycled PMMA particles and their particle size. Five groups were established based on weight/weight (w/w%) concentrations of recycled PMMA:

- Group 1 (Control): 100% virgin PMMA.
- Group 2: 90% virgin PMMA + 10% fine particles.
- Group 3: 80% virgin PMMA + 20% fine particles.
- Group 4: 90% virgin PMMA + 10% whole particles.
- Group 5: 80% virgin PMMA + 20% whole particles.

Recycled PMMA was sourced from milling residuals of Ivotion Base Discs (Ivoclar, Vivadent, Schaan, Liechtenstein). Whole particles were collected directly from the milling machine and used in their collected form without further modification. Fine particles were produced using an Electric Coffee Grinder (ALW-CG10-UK, Cosicosy, China) for 60 s and sieved through a 400-micron mesh for uniform size.

An aluminum mold, custom-designed and milled to produce specimens measuring 66 × 11 × 4 mm³, was utilized for uniformity. The mold was coated with silicone spray to prevent adhesion.

The PMMA mixture was prepared using Pro Base Hot (Ivoclar, Vivadent, Schaan, Liechtenstein) in a powder-to-liquid ratio of 22.5 g:10 ml. The recycled PMMA particles, according to their group allocation, were mixed thoroughly into the PMMA dough. Once the mixture reached the dough stage, it was packed into the mold, sealed, and polymerized in a Polymax pressure polymerizing unit (Dreve Dentamid GmbH, Germany) at 100 °C for 45 min.

The specimens were then cooled, deflasked, and trimmed using an acrylic bur and a metallographic grinding and polishing machine (Tegramin-25, Struers, UK) to achieve the final ISO 178:2019-compliant dimensions of 64 × 10 × 3.3 mm³ for flexural strength testing. Final adjustments were made through sequential sanding with silicon carbide grinding papers (500, 1000, and 1200 grit).

A total of 75 specimens (64 × 10 × 3.3 mm³) were fabricated, with 15 per group. Of these, 10 specimens per group were allocated for flexural strength testing. The remaining five specimens per group were further sectioned using a precision cutting saw (Isomet^®^ 1000, Buehler, Illinois, USA) at the lowest load into 20 smaller samples with a square cross-Sect. (10 × 10 × 3.3 mm³). These were evenly distributed, with 10 specimens per group used for surface hardness testing and 10 for surface roughness testing.

### Mechanical testing

#### Flexural strength

**Fig. 1:**
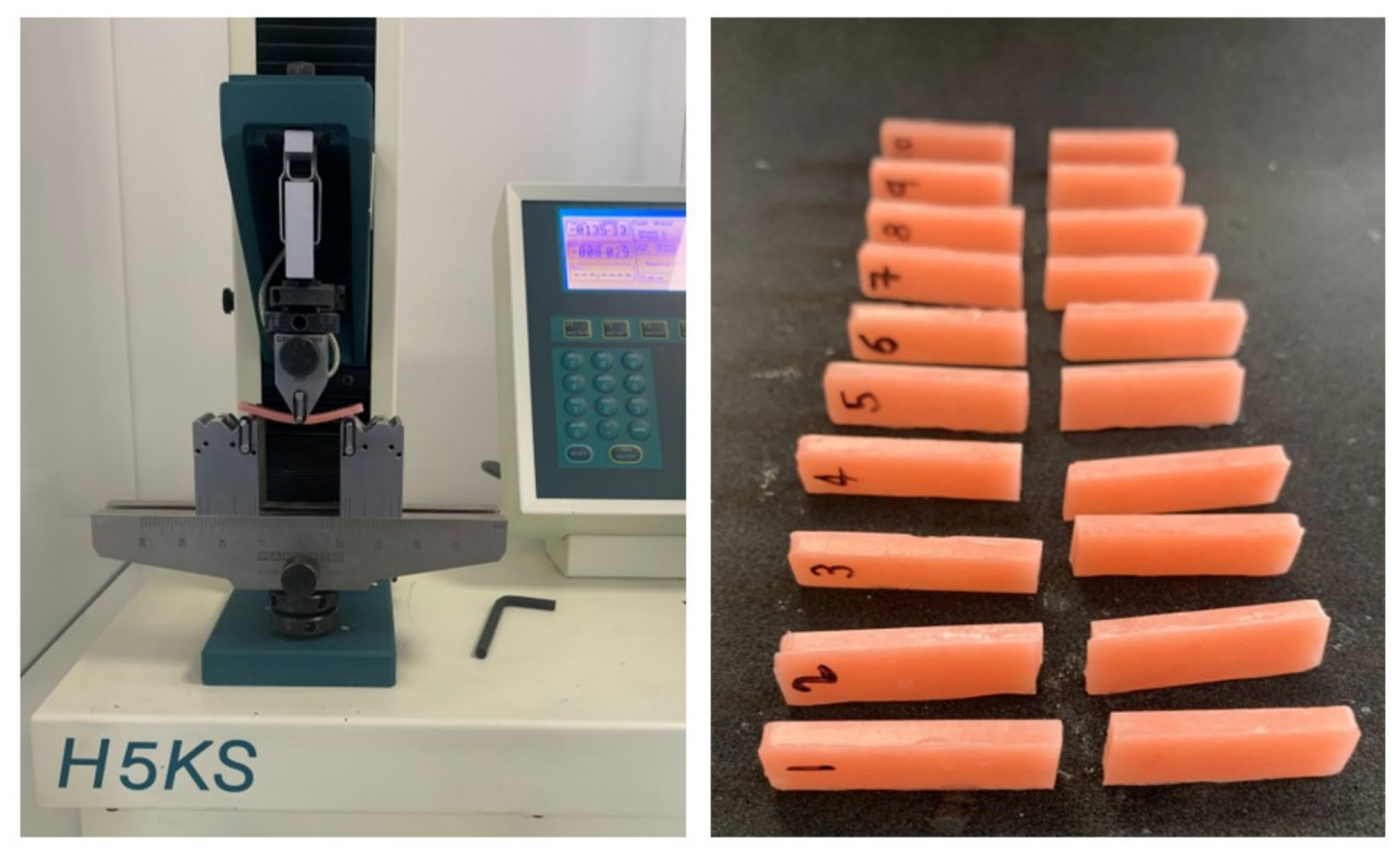
Left: Specimen under flexural load in a three-point bending test using a universal testing machine. Right: Fractured specimens from Group 3 (Fine 20%) after flexural strength testing

Specimens were subjected to three-point bending tests on a universal testing machine (Tinius Olsen H5KS) (Fig. 1). A constant load was applied at a rate of 5 mm/min until fracture. The flexural strength (𝜎) was calculated using:

$$\:{\sigma\:}=\frac{3FL}{{2bh}^{2}}$$

where 𝐹 is the load at fracture (N), 𝐿 is the span length (50 mm), 𝑏 is specimen width (10 ± 0.2 mm), and ℎ is specimen thickness (3.3 ± 0.2 mm).

#### Surface hardness

Surface hardness was assessed using the Vickers hardness test (Duramin-40, Struers). A 300 g load was applied for 15 s with a diamond indenter on square specimens (10 × 10 × 3.3 mm [3]). Each specimen was tested at three points, spaced 1 mm apart, and the Vickers Hardness Number (VHN) was calculated using the formula:

$$\:HVN=1.8544\frac{F}{{d}^{2}}$$

where 𝐹 is the force applied (kgf) and 𝑑 is the mean diagonal length of the indentation.

#### Surface roughness

Surface roughness was measured using a Profilm3D optical profilometer (Filmetrics). Polished specimens were analyzed to determine Ra values by tracing a central line across the specimen surface. Specimens were polished and pumiced for 1 min each prior to testing to ensure uniformity. Surface roughness was assessed on square specimens (10 × 10 × 3.3 mm [3]).

Statistical analysis was conducted using SPSS (IBM Corp., Chicago, IL). Two-way ANOVA and Tukey post hoc tests identified significant differences between groups, with a significance threshold of 𝑝<0.05. Descriptive statistics, including mean, standard deviation, and standard error, were computed using SPSS and Microsoft Excel.

## Results

### Flexural strength

The flexural strength of all groups was within the acceptable ISO 20795-1:2013 range (> 65 MPa), with mean values ranging from 78.69 MPa in Group 4 (Whole 10%) to 96.41 MPa in Group 1 (Control). Statistical analysis using two-way ANOVA revealed significant differences among the groups (𝑝<0.05). Tukey post hoc testing indicated that the control group (Group 1) did not differ significantly from Groups 2 (Fine 10%) or 3 (Fine 20%) (𝑝=0.52 and 𝑝=0.50, respectively). In contrast, significant differences were observed between the control group and Groups 4 (Whole 10%) and 5 (Whole 20%) (𝑝=0.00 and 𝑝=0.02, respectively) (Table 1).

### Surface hardness

Mean surface hardness values ranged from 12.57 VHN in Group 5 (Whole 20%) to 18.73 VHN in Group 3 (Fine 20%). Two-way ANOVA demonstrated significant differences across the groups (𝑝<0.05). Tukey post hoc analysis showed no significant differences between the control group (Group 1) and Groups 2 (Fine 10%) or 3 (Fine 20%) (𝑝=0.96 and 𝑝=0.81, respectively). However, significant differences were observed between the control group and Groups 4 (Whole 10%) and 5 (Whole 20%) (𝑝=0.0066 and 𝑝=0.0004, respectively) (Table 1).

### Surface roughness

Surface roughness values (Ra) were consistent across all groups, ranging from 0.09 μm (Group 1, Control) to 0.13 μm (Group 4, Whole 10%). Statistical analysis using two-way ANOVA showed no significant differences among the groups (𝑝>0.05) (Table 1).

**Table 1:** Combined results for flexural strength, surface hardness, and surface roughness of heat-cured PMMA denture bases with varying percentages and particle sizes of recycled PMMA. Values are presented as mean ± standard deviation (SD) with standard error (SE) in parentheses

Group^1^	Flexural Strength (MPa)	Surface Hardness (VHN)	Surface Roughness (Ra, µm)
	Mean ± SD (SE)	Mean ± SD (SE)	Mean ± SD (SE)
Group 1: Control	96.41 ± 5.00 (1.58)^*a*^	17.67 ± 2.71 (1.21)^*a*^	0.09 ± 0.04 (0.02)
Group 2: Fine 10%	92.52 ± 3.37 (1.07)^*a*^	18.32 ± 0.51 (0.23)^*a*^	0.12 ± 0.04 (0.02)
Group 3: Fine 20%	92.13 ± 5.82 (1.84)^*a*^	18.73 ± 0.41 (0.18)^*a*^	0.10 ± 0.03 (0.01)
Group 4: Whole 10%	78.69 ± 5.18 (1.64)^*b*^	16.33 ± 1.59 (0.71)^*b*^	0.13 ± 0.06 (0.03)
Group 5: Whole 20%	87.84 ± 9.09 (2.87)^*b*^	12.57 ± 1.32 (0.59)^*c*^	0.11 ± 0.04 (0.02)

^1^ Groups sharing the same superscript letter (𝑎,𝑏,𝑐) are not significantly different (𝑝>0.05), while groups with different superscripts indicate significant differences (𝑝<0.05). Statistical analysis was performed using two-way ANOVA followed by Tukey HSD post hoc tests

## Discussion

The study aimed to evaluate the feasibility of incorporating recycled PMMA particles into virgin PMMA denture base mixtures, assessing their impact on flexural strength, surface hardness, and surface roughness. These findings contribute to sustainable solutions for dental materials, addressing environmental and economic challenges associated with PMMA waste.

Flexural strength, a critical property for dental materials, determines their ability to withstand masticatory forces [15]. While fine particles (Groups 2 and 3) maintained flexural strength levels comparable to the control, whole particles (Groups 4 and 5) resulted in reduced performance.

These findings suggest that fine particles enhance interfacial bonding due to their larger surface area-to-volume ratio, which facilitates stress dispersion within the matrix. In contrast, whole particles may create weak points, reducing the material’s ability to withstand stress.

According to a previous study, a gradual decline in mechanical properties was observed as particle size increased, attributed to reduced surface area available for polymer-monomer interaction [16]. In conventional PMMA manufacturing, smaller particles provide a greater surface area, promoting MMA monomer diffusion and polymerization, ultimately leading to stronger interfacial adhesion and improved flexural strength. Conversely, larger particles exhibit weaker bonding with the polymer matrix, increasing the likelihood of stress concentrations and void formation, potentially compromising structural integrity [17].

Similar observations have been made in prior studies that highlighted the detrimental effects of large particle sizes on mechanical properties [14, 18]. The lack of a significant impact from percentage variation in our study further supports the idea that particle size, rather than quantity, is the dominant factor influencing flexural strength. These findings reinforce the importance of particle size control when integrating recycled PMMA into denture base materials to maintain optimal mechanical properties.

Surface hardness reflects a material’s resistance to deformation and wear, which is essential for the longevity of dental prosthetics [19]. The hardness values observed in the control and fine particle groups align with previously reported values for heat-cured PMMA denture base materials [20, 21], indicating that the incorporation of fine recycled PMMA did not negatively impact material performance. However, Group 5 (Whole 20%) demonstrated the lowest hardness, which warrants further discussion. It has been established that polymerization kinetics and porosity levels are influenced by particle size distribution, with larger PMMA particles generally exhibiting better polymerization efficiency and reduced porosity [22]. However, when particles exceed a certain size threshold, their incorporation into the polymer matrix may be compromised, leading to suboptimal material properties. One study suggested that excessively large polymeric particles might become coated with the polymerizing monomer rather than fully integrating into the polymer network, ultimately reducing hardness [23]. In the present study, whole particles exceeded 400 microns, and previous research has shown that larger particles (> 400 microns) exhibit lower monomer conversion rates (< 85%), which may negatively impact final polymerization outcomes and mechanical performance [17]. This could explain the significant reduction in hardness observed in the group 5, where a higher concentration of oversized particles might have disrupted the polymer matrix, leading to lower hardness values.

Surface roughness significantly influences both the functional and aesthetic performance of dental materials, particularly by affecting bacterial adhesion and plaque accumulation [24]. In this study, no significant differences in surface roughness were observed among the groups, indicating that neither the particle size nor the concentration of recycled PMMA influenced this property. Although the Control group exhibited the lowest Ra values, these differences were statistically insignificant, with all mean Ra values ranging from 0.09 μm to 0.13 μm, which is well below the clinically acceptable threshold of 0.2 μm for denture base materials [25]. Notably, these values are comparable to, or in some cases slightly more favourable than, those reported in previous research on enhanced PMMA formulations [26, 27]. The uniformity observed across all groups supports the feasibility of integrating recycled PMMA and provides reassurance regarding its aesthetic and functional suitability for clinical applications.

The results highlight that particle size significantly influenced specific mechanical properties, with fine particles maintaining flexural strength and enhancing surface hardness compared to whole particles. However, percentage variation had a limited impact on flexural strength and contributed to improved hardness only in fine particle groups. Surface roughness remained unaffected by both factors, demonstrating the resilience of PMMA’s surface properties despite material modifications.

This study provides evidence for the sustainable use of recycled PMMA in dental materials, offering a viable solution to reduce waste and enhance environmental stewardship. The findings suggest that fine particle integration is key to maintaining or improving specific mechanical properties. However, the inability to enhance flexural strength beyond control levels highlights the need for further optimization of material formulations. The selection of 10% and 20% recycled PMMA concentrations was based on an initial estimation by the researchers, aiming to balance material integrity with sustainability. While these concentrations provided insight into feasibility, future studies should explore a broader range of concentrations to determine the optimal ratio for maximizing mechanical properties while maintaining sustainability benefits. Additionally, the study focused exclusively on heat-cured PMMA and did not evaluate long-term performance, biocompatibility, or clinical outcomes. Another limitation is that whole particles were used in their collected form without detailed particle size distribution analysis, and their shape was not systematically characterized. Future research should address these limitations, exploring broader material types, advanced testing methods, and cost-effectiveness analyses.

## Conclusions

The incorporation of recycled PMMA particles into virgin PMMA mixtures shows promise as a sustainable approach to dental material development. While the inclusion of fine particles maintained acceptable flexural strength levels comparable to the control, whole particles resulted in reduced performance. Additionally, fine particles significantly enhanced surface hardness without compromising surface roughness, demonstrating their potential for improving specific mechanical properties. These findings highlight the feasibility of recycling PMMA waste for use in denture base materials, aligning with sustainability goals and reducing environmental impact. However, further research is needed to optimize material formulations and fully assess their long-term clinical performance and economic viability.

## Data Availability

The datasets used and/or analysed during the current study are available from the corresponding author on reasonable request.
